# Biotic and abiotic properties mediating sediment microbial diversity and function in a river–lake continuum

**DOI:** 10.3389/fmicb.2024.1479670

**Published:** 2024-10-21

**Authors:** Yabing Gu, Delong Meng, Zhenghua Liu, Min Zhang, Zhaoyue Yang, Huaqun Yin, Yanjie Liang, Nengwen Xiao

**Affiliations:** ^1^School of Metallurgy and Environment, Central South University, Changsha, China; ^2^State Key Laboratory of Environmental Criteria and Risk Assessment, Chinese Research Academy of Environmental Sciences, Beijing, China; ^3^School of Minerals Processing and Bioengineering, Central South University, Changsha, China

**Keywords:** river–lake continuum, environmental factor, macrozoobenthos, microbial ecology, functional potential

## Abstract

A river–lake system plays an important role in water management by providing long-term and frequent water diversions. However, hydrological connectivity in the system can have a profound effect on sediment microbial communities through pH, nutrient concentrations, and benthos invertebrates. Consequently, identifying the key environmental factors and their driving mechanisms is vital for microbial adaptation strategies to extreme environments. In this study, we analyzed the significant difference in sediment bacterial and fungal community structures and diversity indices among Dongting Lake and its tributary rivers, which worked as a typical river-connected lake ecosystem. There were significant differences in biotic and abiotic environments in the sediment habitats of Dongting Lake and its tributary rivers. Random forest analysis revealed that pH and *Mollusca* were found to be the most important abiotic and biotic variables for predicting both bacterial and fungal community structures, respectively. The beta diversity decomposition analyses showed that the bacterial and fungal community compositional dissimilarities among different sections were dominated by species replacement processes, with more than half of the OTUs in each section being unique. Notably, both biotic and abiotic factors affected the number and the relative abundance of these bacterial and fungal unique OTUs, leading to changes in community composition. *Mollusca*, pH, TP, NO_3_-N, and NH_4_-N were negatively related to the relative abundance of *Actinobacteria, Acidobacteria, Gemmatimonadetes, Planctomycetes*, and *Ascomycota*, while *Annelida* and ORP were positively related to the relative abundance of *Actinobacteria* and *Gemmatimonadetes*. Additionally, PICRUSt analysis revealed that the functional dissimilarity among lakes and rivers was strengthened in unique species compared to all species in bacterial and fungal communities, and the changes of functional types helped to improve the habitat environment in the main Dongting Lake and promote the process of microbial growth. From our results, the role of macrozoobenthos and physicochemical characteristics in driving the sediment microbial community spatial variations became clear, which contributed to further understanding of the river–lake ecosystem.

## 1 Introduction

Freshwater ecosystems play an important role in providing habitats for a variety of unique species, supporting key ecosystem functions, and providing essential ecosystem services (Cid et al., 2021). However, aquatic biodiversity in freshwater ecosystems has faced significant threats and dramatic declines globally due to increasing anthropogenic disturbances, such as hydrological alteration, habitat fragmentation, overexploitation, and water pollution (Vörösmarty et al., 2010; Burlakova et al., 2011; Geist, 2011; Reis et al., 2019). Hydrological connectivity is vital to sustaining hydrological and ecological processes between the river and adjacent waterbodies such as secondary channels, lakes, and wetlands (Amoros and Bornette, 2002). First, hydrological connectivity can have a profound effect on aquatic microbial communities through pH, organic substrate availability, and nutrient concentrations (Argiroff et al., 2016). Then, a high degree of hydrological connectivity supports a high level of species richness (Heino et al., 2014), including fish, macroinvertebrates, and zooplankton (Leigh and Sheldon, 2009; Shen and Liu, 2021; Zheng et al., 2022) because of the high dispersal rate. The rivers and lakes that were connected can be understood as a continuum, particularly in studies of aquatic microbial ecology (Tang et al., 2020). At present, there is a knowledge gap concerning the relationships between microbial communities in lakes and their input rivers in the river–lake continuum. The extent to which input rivers influence microbial communities in their downstream lakes has yet to be thoroughly investigated. This limited our understanding of aquatic ecosystem structure and function, preventing effective predictions of lake ecosystem responses to environmental change.

Benthos macroinvertebrates serve as excellent indicators of aquatic ecosystem structure and function because they contribute to biogeochemical cycling such as carbon, nitrogen, and sulfur (Dos Santos et al., 2011; Elgueta et al., 2021). For example, a high *Tubificidae* and *Chironomus larvae* density can influence the release of nitrogen and sulfur, which can accelerate the decomposition rate of organic detritus, as well as regulate the exchange of matter between sediments and water (Fukuhara and Sakamoto, 1987). *Neotrypaea californiensis*, another globally distributed macrofauna, transports oxygenated and anoxic water in sediments, causing oxic-anoxic oscillations (Liu et al., 2012). In spite of the direct impact macroinvertebrates have on sediment biogeochemical cycling, it has been suggested macroinvertebrates contribute more to sediment processes through their diverse direct and indirect interaction with sediment microbes (Grandy et al., 2016; Petersen and Osvatic, 2018). Caddisflies created niches enriched with denitrifiers to engineer the stream microbiome through low-oxygen niche creation (Bertagnolli et al., 2023). The presence of *Naidid worms* increased the relative abundance of *Betaproteobacteria* and decreased the relative abundance of *Chlorobi* in the surface sediment (Zeng et al., 2014). In addition to facilitating the vertical and horizontal distribution of the elements along with the sediment, various physical activities of macrozoobenthos disrupt natural sediment architecture and affect benthic microbial assemblage by reshuffling substratum during various physical activities (Adámek and Maršálek, 2012; Chakraborty et al., 2022).

In the past few decades, researchers have been interested in quantifying the influence of macrozoobenthos on sediment microbes, yet most studies on microbial responses to invertebrates have focused on single species of invertebrates, such as earthworms, isopods, or millipedes (Maraun and Scheu, 1996; Dempsey et al., 2011; Crowther et al., 2015). Researchers studying the relationship between macroinvertebrate-driven changes in microbial community composition and functions have found that macroinvertebrates can significantly impact microbial biomass and have contrasting effects on fungal and bacterial communities (Chang et al., 2017). However, in natural environments, multiple macroinvertebrate taxa are simultaneously influencing microbial communities through different pathways. Therefore, accurately measuring microbial responses to diverse macroinvertebrates at the community level is essential for comprehending the role of macrozoobenthos in shaping soil microbial communities.

Dongting Lake is the first large lake in the area downstream of the Yangtze River basin, characterized by a clear seasonal rhythm consisting of a dry season and a flood season with a highly fluctuating water level (Liang et al., 2018). As a representative river-connected lake, Dongting Lake receives water from four primary tributaries (Xiangjiang River, Zishui River, Yuanjiang River, and Lishui River) and has three outfalls (Songzi, Taiping, and Ouchi). Inflow water is regulated by Dongting Lake, which discharges into the Yangtze River. Thus, Dongting Lake is an inland lake with a flood channel and has a complex river–lake relationship with its inflow rivers. It is an ideal site at which to identify the effects of tributary rivers on the microbial communities in the main lake. Rivers and lakes can only generally be understood as a continuum because rivers change constantly as they move from headwaters to downstream lakes (Ylla et al., 2013). Previous studies have found that the discharge from the upstream tributaries in the lake basin and the water recharge via the connection to the Yangtze River outside the basin would influence the nutrient concentration of water and sediment in Dongting Lake directly (Tian et al., 2017). Meanwhile, microorganisms travel from one habitat to another along with particulate organic matter as a result of river and lake connectivity (Tang et al., 2020). Thus, a reorganization of microbial communities is created with the influences of environmental factors. It is crucial to take into account the entire network of interconnected water bodies (such as rivers and lakes) in order to comprehensively grasp the principles governing the assembly of microbial communities in aquatic ecosystems.

While the temporal and spatial variations of microbial community composition in Dongting Lake and its upstream tributaries have been examined recently, little is known about what and how the biotic and abiotic variables influenced the microbial community under the river–lake continuum. In this study, the Dongting Lake watershed worked as a typical river-connected lake ecosystem to investigate the microbial diversity, community composition, and functional types in both tributary river and lake systems simultaneously. We hypothesized that (i) the main lake has higher species diversity than the tributary rivers because of more unique species from various tributary rivers; (ii) biotic (macrozoobenthos) and abiotic factors both help drive the microbial community pattern in sediment; and (iii) the functional types would be influenced with the replacement of unique OTUs. This study is the first attempt to explore the effects of macrozoobenthos on microbial communities at the community level in a river–lake continuum. Therefore, it makes a fundamental contribution to the mechanism understanding of a protective microbial ecology in the river–lake continuum.

## 2 Materials and methods

### 2.1 Site description and sampling of sediment samples

**Figure 1:**
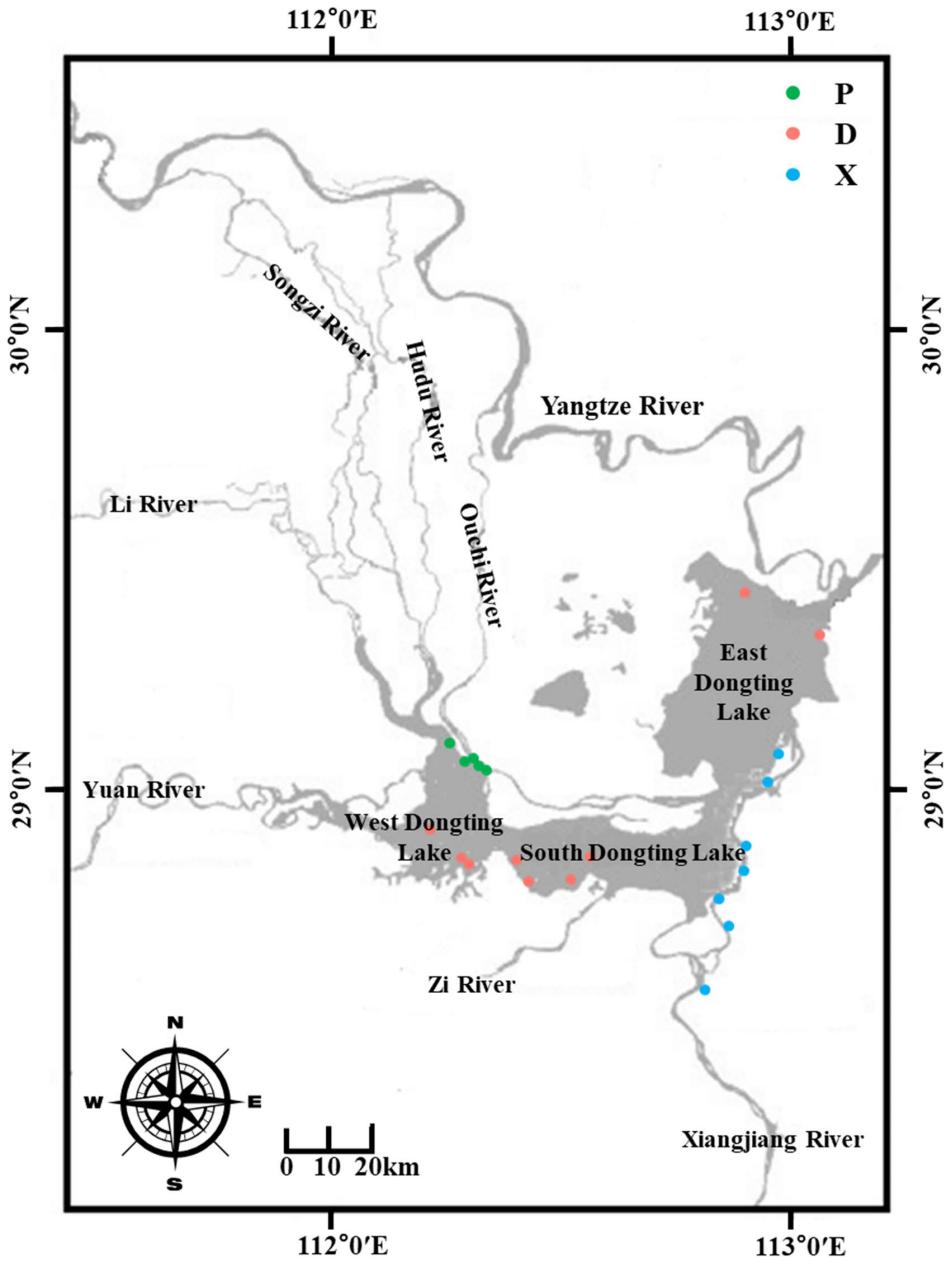
Localization of the sediment sampling stations D, P, and X located around the Dongting Lake basin. D: samples from the main Dongting Lake including West Dongting Lake, South Dongting Lake, and East Dongting Lake. P: samples from the pooling from three outfalls through Songzi River, Hudu River, and Ouchi River. X: samples from the Xiangjiang River.

This study was conducted in the Dongting Lake basin, China, during the flood season (from July to September 2018). Dongting Lake receives recharge from the three outfalls in the northwest from the Yangtze River and Xiangjiang in the south. To measure how and the extent to which the main lake can be influenced by its tributary rivers, surface sediment samples from the main lake (D) and tributary lakes including Xiangjiang River (X) and pooling from three outfalls (P) were collected as described in Figure 1. The longitude and latitude of each sampling site are described in Supplementary Table S1. Xiangjiang River originates from the Longmenjie district in Lingui County of Guangxi Zhuang Autonomous Region and flows into the Dongting Lake from south to north. Water diverted from the Yangtze River flowing through the three outfalls, including Songzi River, Huduhe River, and Ouchi River, gathers in the western part of Dongting Lake and flows into the main lake. Nine samples were collected from each section, totaling 27 sediment samples. Each sediment sample was divided into two parts, one of which was packed in a 50-mL sterile centrifuge tube and stored at −80°C for microbial community analyses in the laboratory. The other was kept in sterile plastic bags and air-dried for the analysis of physical and chemical properties. Meanwhile, the biology investigation of benthic macro-invertebrates at corresponding sampling sites was conducted by the Chinese Research Academy of Environmental Sciences. A grab sampler was used to collect samples from a depth of 10 cm from the top of the sediment surface, and the specimens were identified and counted in the laboratory.

### 2.2 Sediment chemical composition analysis

After drying in air and filtering through a 2-mm sieve, the values of pH, oxidation–reduction potential (ORP), organic matter (OM), total nitrogen (TN), total phosphorus (TP), organic phosphorus (OP), ammoniacal nitrogen (NH_4_^+^-N), and nitrate nitrogen (NO_3_^−^-N) for all sediment samples were tested in the laboratory of Nanjing Institute of Geography and Limnology, Chinese Academy of Sciences. The values of pH and oxidation–reduction potential (ORP) were measured using a digital pH/Ec meter in a sediment:water ratio of 1:2.5 (w/v) with dried sediment samples. OM content was determined using the loss-on-ignition method on oven-dry soil (Nelson and Sommers, 1996). TN, NH_4_^+^-N, and NO_3_^−^-N were measured using the Kjeldahl nitrogen method (D'Elia et al., 1977). TP and OP were measured using the molybdenum-blue method (Eisenreich et al., 1975).

### 2.3 Sediment DNA extraction, PCR amplification, and high-throughput sequencing

Total sediment DNA extraction followed the DNA extraction method of the FastDNA Kit, and then the DNA quality and quantity were evaluated using a NanoDrop ND-2000 spectrophotometer (ND-1000 Spectrophotometer, America). The V3–V4 region of the 16S rRNA gene was amplified using primer pairs 341F (5′-CCTACGGGNGGCWGCAG-3′) and 805R (5′-GACTACHVGGGTATCTAATCC-3′) for the bacterial community. The internal transcribed spacer (ITS) ribosomal region was amplified using the primers fITS7 (5′-GTGARTCATCGAATCTTTG-3′) and ITS4 (5′-TCCTCCGCTTATTGATATGC-3′) for the fungal community. Next-generation sequencing library preparations and high-throughput sequencing were conducted at LC Sciences (Hangzhou, China) with an Illumina MiSeq platform (Illumina, San Diego, United States). The 16S rRNA gene and ITS gene sequences were submitted to the NCBI SRA database, and the project numbers were PRJNA801141 and PRJNA801493, respectively.

All raw sequences were uploaded and processed in the galaxy pipeline^1^ constructed by Dr. Zhou (Institute for Environmental Genomics, University of Oklahoma). Clean reads after trimming barcodes and primers were merged using Flash (Magoc and Salzberg, 2011). An operational taxonomic unit (OTU) table was generated based on the 97% similarity threshold using UPARSE (Edgar, 2013). The SILVA (Quast et al., 2013) and Unite (Abarenkov et al., 2010) databases were used as the reference databases for bacterial and fungal taxonomic assignments, respectively. After trimming the singleton and subsampling to the minimum reads per sample, sequences were reduced to 21,000 bacterial reads and 50,000 fungal reads, respectively.

### 2.4 Analysis of the amplicon data

Microbial and zoobenthos community diversity indices, including Shannon index, Species richness, and Pielous’ evenness, were calculated using *vegan* package (Oksanen et al., 2013). Differences among sampling sections were determined using ANOVA followed by *Fisher’s* test. Principal coordinates analysis (PCoA) based on Bray–Curtis distance was performed to evaluate the similarity of microbial and zoobenthos communities among different sections. The analysis of similarity (ANOSIM), multi-response permutation procedure (MRPP), and permutational multivariate analysis of variance (ADONIS) methods were utilized to detect significant differences among sections. Compositional dissimilarities among sections were partitioned into replacement and richness difference components (Podani family, Jaccard dissimilarities) using *adespatial* package (Dray, 2016). To test the relative importance of biotic and abiotic variables in driving bacterial and fungal community structures, random forest analysis was performed using *randomForest* package (Liaw and Wiener, 2002). The regression was conducted using the ‘randomForest’ function and the importance of variables was determined by the value of %IncMSE (increased in mean squared error) calculated by the ‘importance’ function. We identified the unique OTUs among different sections through a Venn diagram using *vennDiagram* package (Chen and Boutros, 2011). The relationships among variables and the number and relative abundance of unique OTUs were measured using the linear regression analysis. A heatmap for unique OTUs at the phylum level was established using the *pheatmap* package with a complete linkage clustering method and Euclidean distance (Kolde and Kolde, 2015). Relationships between variables and phylum with significant differences among different sections were also measured using the linear regression analysis and visualized with a heatmap.

Functions were predicted based on bacterial and fungal taxa through PICRUSt2 Analysis using the OmicStudio Analysis^2^ (Douglas et al., 2020). The KEGG database and MetaCyc database were used to predict the functional pathways of each bacterial and fungal community in the sediment, respectively (Kanehisa et al., 2017; Caspi et al., 2018). PCoA based on Bray–Curtis distance was performed to evaluate the similarity of function types among different sections for all and unique OTUs. ADONIS method was utilized to detect significant differences among sections. Function types showed significant differences among sections were determined through the least significant difference (LSD) test and visualized with a bar plot.

## 3 Results

### 3.1 Comparison of bacterial, fungal, and zoobenthos community diversities among main lake and tributary rivers

**Figure 2:**
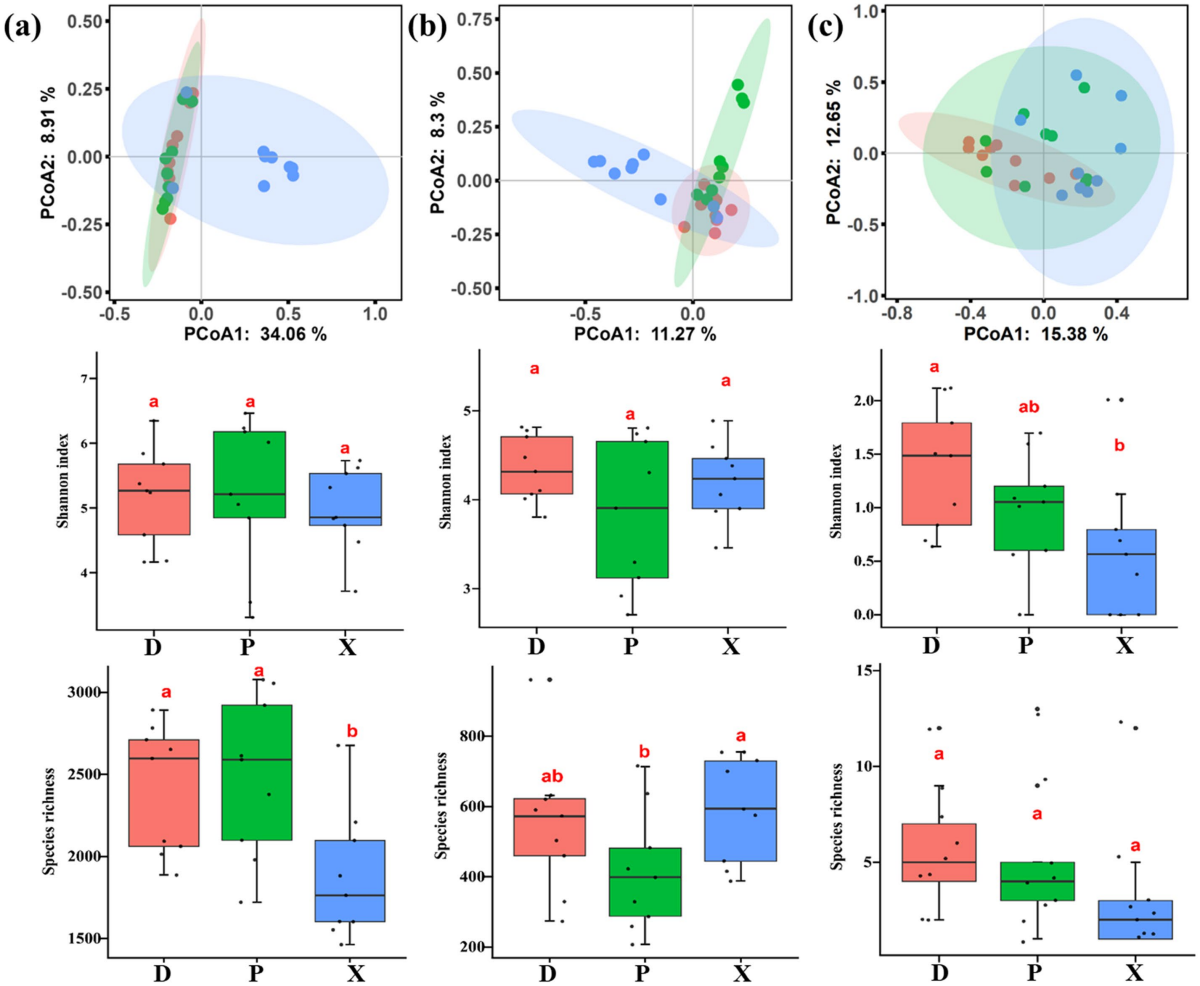
PCoA analysis, Shannon index, and species richness of bacterial (a), fungal (b), and benthos macroinvertebrate (c) communities in Dongting Lake (D) and its tributary rivers, including Xiangjiang River (X) and pooling from three outfalls (P). Boxes with different lowercase letters were significantly different (*p* < 0.05).

Bacterial, fungal, and macrozoobenthos community diversities were analyzed, revealing significant heterogeneity among main and tributary lakes. PCoA plots indicated that there were significant separations between D and X, and between P and X (Figures 2a,b), but there was no significant separation between D and P. Additionally, dissimilarity analysis including MRPP, ANOSIM, and ADONIS all found significant differences in bacterial community structures between D and X, and between P and X (Bray–Curtis distance, *p* < 0.01) (Table 1). Significant differences were also found in fungal community structures among D, P, and X (Bray–Curtis distance, *p* < 0.01). The PCoA plot and dissimilarity analysis both showed significant differences for macrozoobenthos community structures among D, P, and X (Bray–Curtis distance, *p* < 0.05) (Figure 2c). According to the result of MRPP, the community structure dissimilarity among D, P, and X was highest for macroinvertebrate community (R = 0.908), followed by fungal community (R = 0.871), and bacterial community (R = 0.670). The species richness of the bacterial community in D and P was significantly higher than that in X, the species richness of the fungal community in P was significantly lower than that in X, and the Shannon index of zoobenthos community in X was significantly lower than that in D (ANOVA, *p* < 0.05).

**Table 1:** Sediment bacterial (B), fungal (F), and zoobenthos (Z) community dissimilarity analysis among the main lake (D) and tributary lakes including Xiangjiang River (X) and pooling from three outfalls (P) through MRPP, ANOSIM, and ADONIS based on Bray–Curtis distance.

Type	Treatment	MRPP		ANOSIM		ADONIS	
		R	p	R	p	F	p
B	All	**0.670**	**0.001**	**0.436**	**0.001**	**4.232**	**0.001**
	DvsL	0.631	0.070	0.090	0.101	1.594	0.073
	DvsX	0.703	**0.002**	0.631	**0.001**	4.881	**0.001**
	LvsX	0.677	**0.001**	0.682	**0.001**	5.789	**0.001**
F	All	0.871	**0.001**	0.395	**0.001**	2.194	**0.001**
	DvsL	0.858	**0.001**	0.328	**0.001**	2.019	**0.002**
	DvsX	0.872	**0.001**	0.446	**0.002**	2.280	**0.004**
	LvsX	0.883	**0.001**	0.450	**0.001**	2.274	**0.001**
Z	All	0.908	**0.001**	0.205	**0.001**	1.786	**0.001**
	DvsL	0.887	**0.031**	0.183	**0.008**	1.675	**0.025**
	DvsX	0.896	**0.001**	0.294	**0.001**	2.349	**0.001**
	LvsX	0.942	0.145	0.153	**0.035**	1.371	0.098

The bold words indicate statistically significant differences among sections (*p* < 0.05).

### 3.2 The distribution of macrozoobenthos community composition among main and tributary rivers

**Figure 3:**
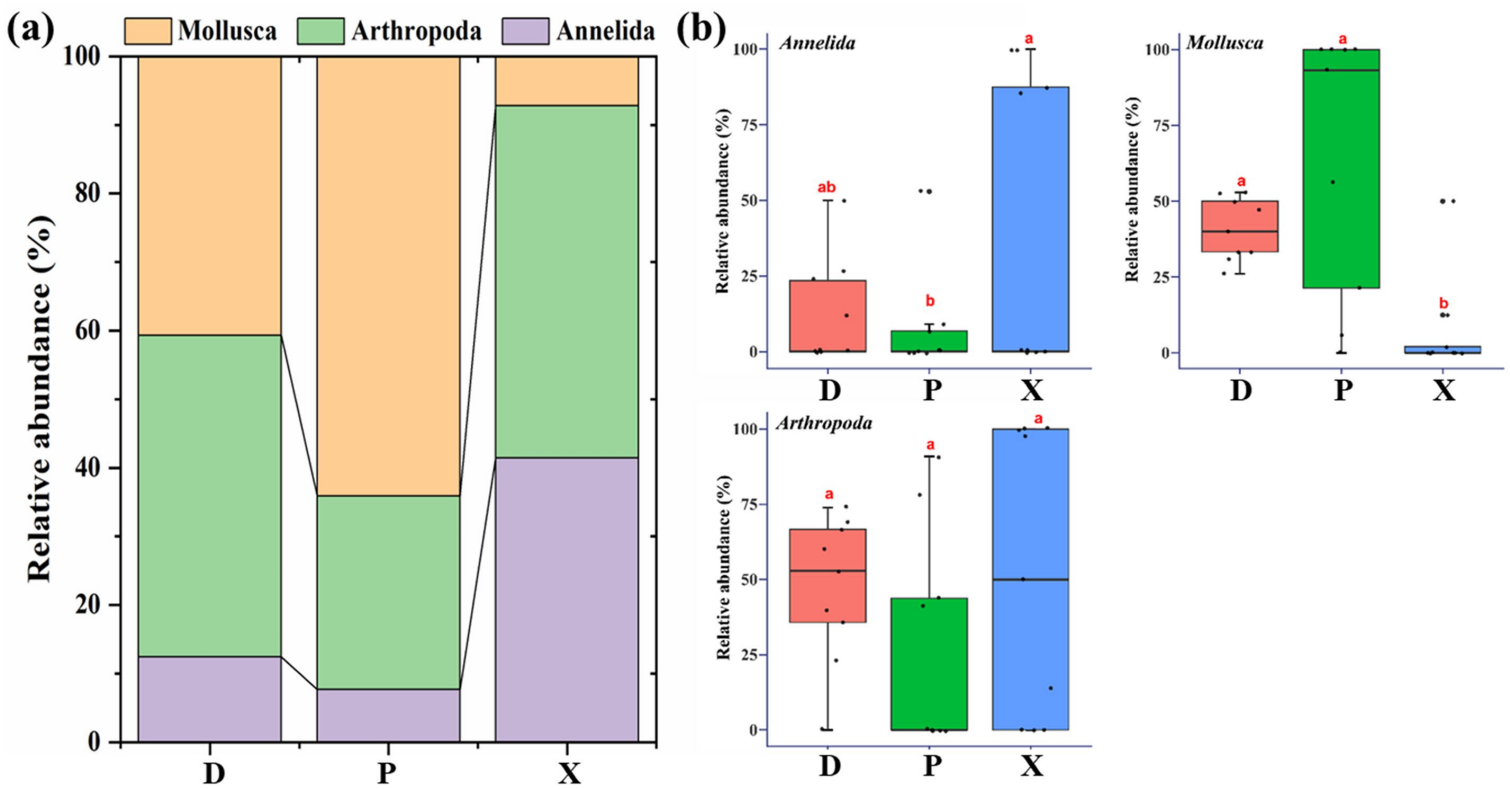
Macrozoobenthos community composition in Dongting Lake (D) and its tributary rivers, including Xiangjiang River (X) and pooling from three outfalls (P). (a) The stacked bar chart of macrozoobenthos communities in three sampling sections at the phylum level. (b) ANOVA analysis for the relative abundance of macrozoobenthos at the phylum level among different sampling sections. Boxes with different letters are significantly different (*p* < 0.05).

Macrozoobenthos communities in D, P, and X consisted of three phyla, including *Mollusca*, *Arthropoda*, and *Annelida* (Figure 3a). *Mollusca* dominated the community composition of P (64.08%), and it was significantly higher in D (40.66%) and P than that in X (7.17%) (ANOVA, *p* < 0.05) (Figure 3b). *Arthropoda* was the dominant phyla of D and X (D, 46.89%; P, 28.27%; X, 51.36%), and it showed no significant difference among D, P, and X. For *Annelida*, its relative abundance in X was significantly higher than that in P (D, 12.46%; P, 7.65%; X, 41.47%).

### 3.3 Contributions of biotic and abiotic factors on microbial community structures

Differences in abiotic environmental factors among main and tributary lakes were evaluated and analyzed according to one-way ANOVA and Fisher’s least significant difference (Table 2). The values of pH were significantly higher in P and D than in X (ANOVA, *p* < 0.05), and the values of ORP were significantly lower in P and D than in X (ANOVA, *p* < 0.05). The content of OM in D was significantly higher than that in P (ANOVA, *p* < 0.05). The contents of NO_3_^−^-N and NH_4_^+^-N in D were significantly higher than that in X (ANOVA, *p* < 0.05). The content of TP in P was significantly higher than that in X (ANOVA, *p* < 0.05). Overall, the environmental factors of the main lake showed significant heterogeneity compared to tributary lakes, and the main lake had higher nutrient contents than tributary lakes.

**Table 2:** ANOVA of sediment physical and chemical properties among different sections based on Fisher’s protected LSD test.

Site	D	L	X
OM	**2.256 ± 1.182a**	**1.374 ± 0.618b**	**1.857 ± 0.796ab**
TN	1088.9 ± 670.1a	840.3 ± 239.0a	805.2 ± 401.6a
TP	**377.3 ± 90.5ab**	**427.4 ± 93.9a**	**292.6 ± 131.9b**
OP	108.65 ± 66.25a	130.16 ± 92.04a	95.62 ± 59.06a
NO^3−^-N	**38.094 ± 13.935a**	**29.646 ± 5.393ab**	**26.591 ± 5.657b**
NH^4+^-N	**26.252 ± 6.849a**	**22.064 ± 4.366ab**	**18.897 ± 4.810b**
ORP	**163.89 ± 35.45b**	**159.11 ± 28.32b**	**219.67 ± 38.71a**
pH	**7.958 ± 0.242a**	**8.114 ± 0.120a**	**6.749 ± 0.815b**

Data are means ± standard errors. Different letters and bold words indicate statistically significant differences among sections (*p* < 0.05). OM, organic matter (mg/kg); TN, total nitrogen (mg/kg); TP, total phosphorus (mg/kg); OP, organic phosphorus (mg/kg); NO_3_^−^-N, nitrate nitrogen (mg/kg); NH_4_^+^-N, ammonium nitrogen (mg/kg); ORP, oxidation–reduction potential (mV).

**Figure 4:**
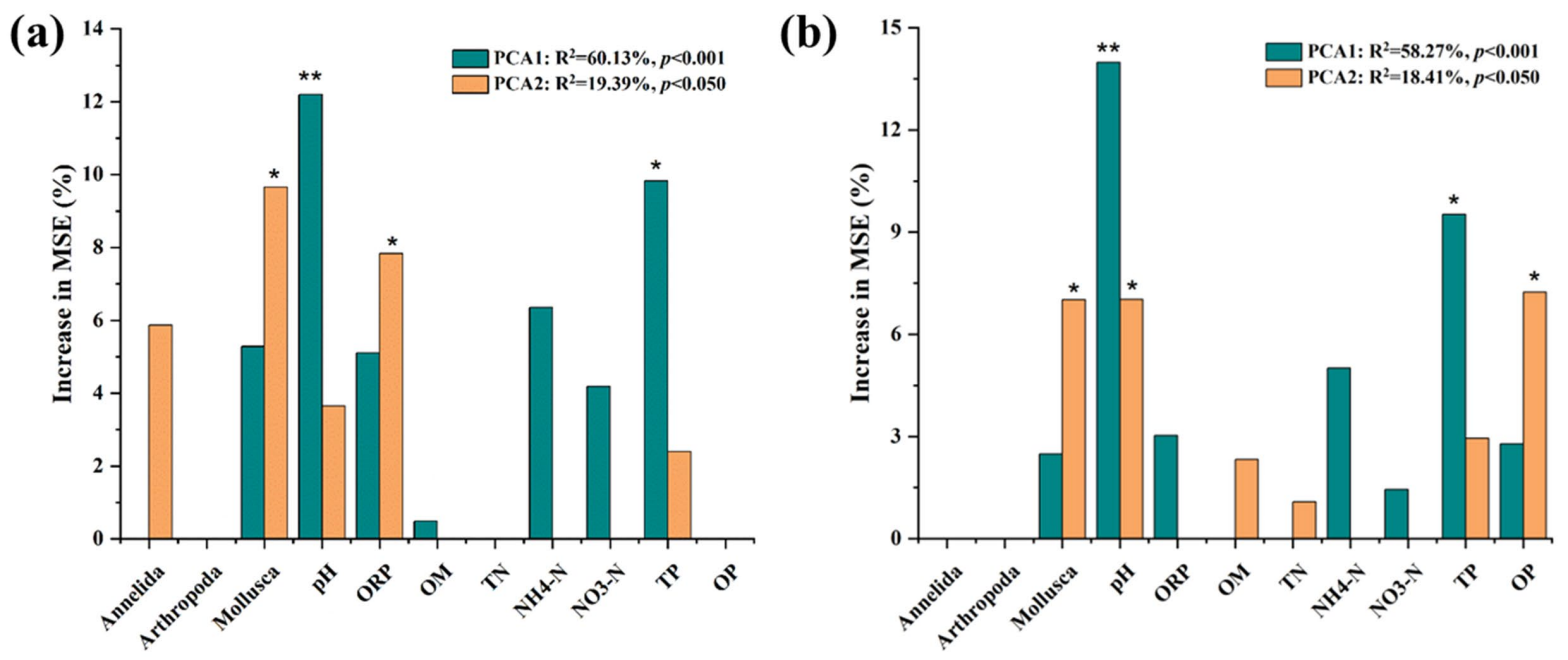
Random forest mean predictor importance (% of increase in mean square error) of abiotic variables and biotic variables studied as predictors of the bacterial (a) and fungal (b) community structures in this study. Abiotic variables meant sediment properties including pH, ORP, OM, TN, NH_4_^+^-N, NO_3_^−^-N, TP, and OP. Biotic variables meant the relative abundance of benthos macroorganisms including *Mollusca*, *Arthropoda*, and *Annelida*. Microbial community metrics were indicated by PCoA1 and PCoA2 values based on Bray–Curtis distance. This accuracy importance measure was computed for each tree and averaged over the forest (5,000 trees). MSE% values are used to estimate the importance of variables, with higher values indicating more significant predictors. Significance levels are as follows: *p < 0.05 and ** *p* < 0.01.

Random forest analysis was conducted to disentangle the potential main drivers of bacterial and fungal community structures in Dongting Lake (Figure 4). The value of pH was found to be the most important abiotic variable for predicting the bacterial community structure (*p* < 0.01), followed by TP and ORP (*p* < 0.05). The abundance of *Mollusca* was the significant biotic factor for bacterial community structure (*p* < 0.05). The value of pH was also the most important abiotic variable for predicting the fungal community structure (*p* < 0.01), followed by TP and OP (*p* < 0.05). The abundance of *Mollusca* was also an important biotic factor for fungal community structure (*p* < 0.05). Compared to the bacterial community, OP showed significant importance in structuring the fungal community, but ORP showed no significant role.

**Figure 5:**
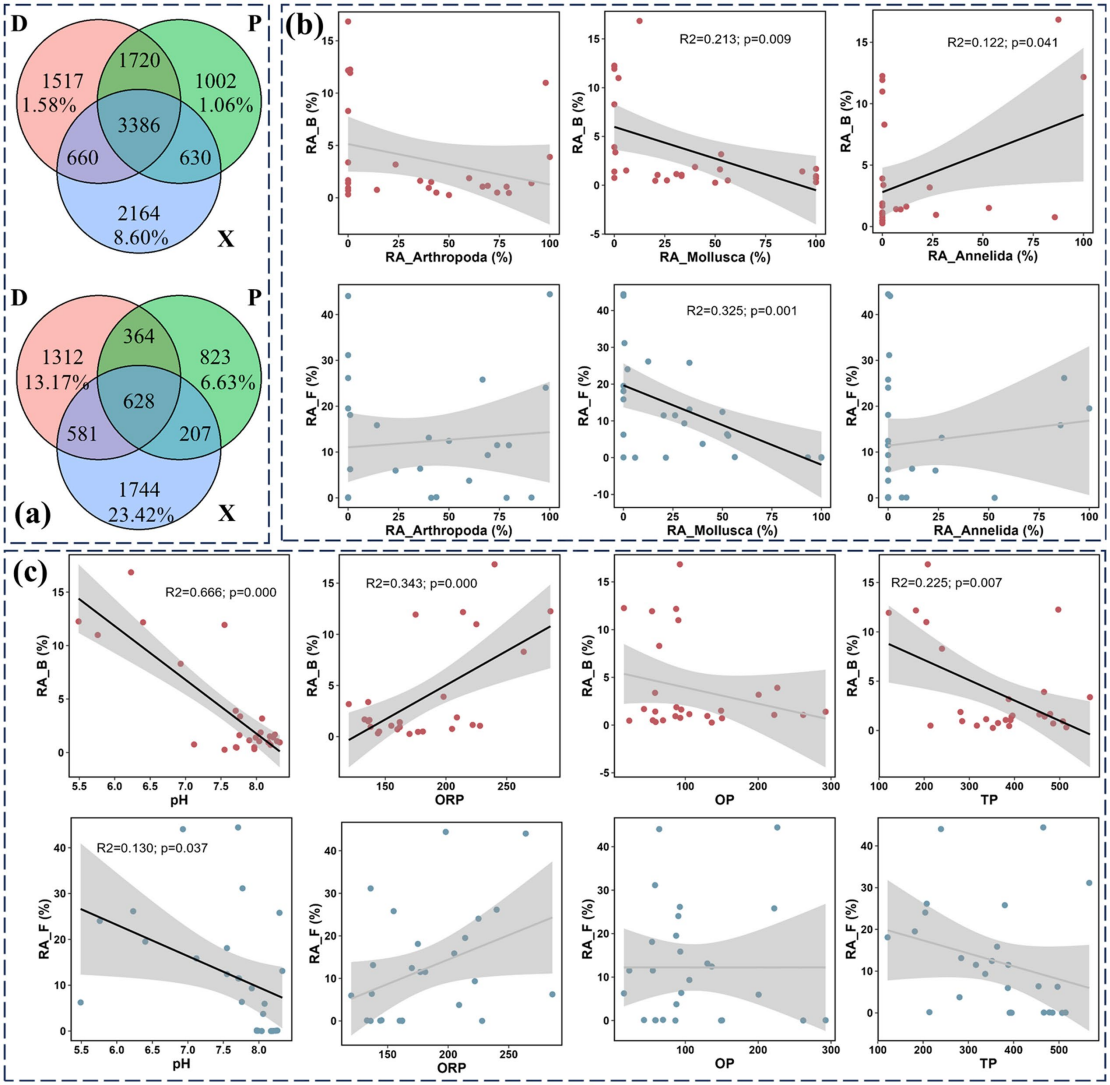
Microbial community composition analysis at the OTU level. Venn diagram showing the differences of sediment bacterial and fungal OTU numbers among main Dongting Lake (D), Xiangjiang River (X), and pooling from three outfalls (P) (a). The relative abundance of unique OTUs was also marked. The linear regression analysis was conducted between the relative abundance of unique microbial OTUs and the biotic variables (the relative abundance of benthos macroorganisms including *Mollusca*, *Arthropoda*, and *Annelida*) (b). The linear regression analysis was conducted between the relative abundance of unique microbial OTUs and the abiotic variables (pH, ORP, TP, and OP) (c). R^2^ is the coefficient of determination and reflects the fitting degree of the regression equation. *p*-value means the significance of linear regression.

The beta diversity decomposition analyses showed that bacterial and fungal community compositional dissimilarities among all study sites were dominated by species replacement processes (contributed 78.43 and 73.64% for bacterial and fungal beta diversity, respectively), while richness difference processes only contributed 21.57 and 26.36% on average. The Venn diagram analysis in Figure 5a revealed that 3,384 and 628 OTUs were shared in bacterial and fungal communities, respectively. Meanwhile, 1,517 (20.81% for OTU number and 1.58% for their total relative abundance), 1,002 (14.88 and 1.06%), and 2,164 (31.63 and 8.60%) unique bacterial OTUs were found in D, P, and X, respectively. Furthermore, 1,312 (45.48% for OTU number and 13.17% for their total relative abundance), 823 (40.70 and 6.63%), and 1744 (55.19 and 23.42%) unique fungal OTUs were found in D, P, and X, respectively. Additionally, the linear regression analysis detected a strong and negative association between the relative abundances of the total unique OTUs and *Mollusca* for bacterial (R^2^ = 0.213) and fungal (R^2^ = 0.325) communities (Figure 5b). The relative abundance of total bacterial unique OTUs was also significantly and positively correlated with *Annelida* (R^2^ = 0.122). The linear regression analysis also identified decreased relative abundances of total bacterial and fungal unique OTUs along with increased pH values (R^2^_B = 0.666, R^2^_F = 0.130) (Figure 5c). The relative abundances of total bacterial unique OTUs showed positive and negative correlations with ORP (R^2^ = 0.343) and TP (R^2^ = 0.225), respectively.

**Figure 6:**
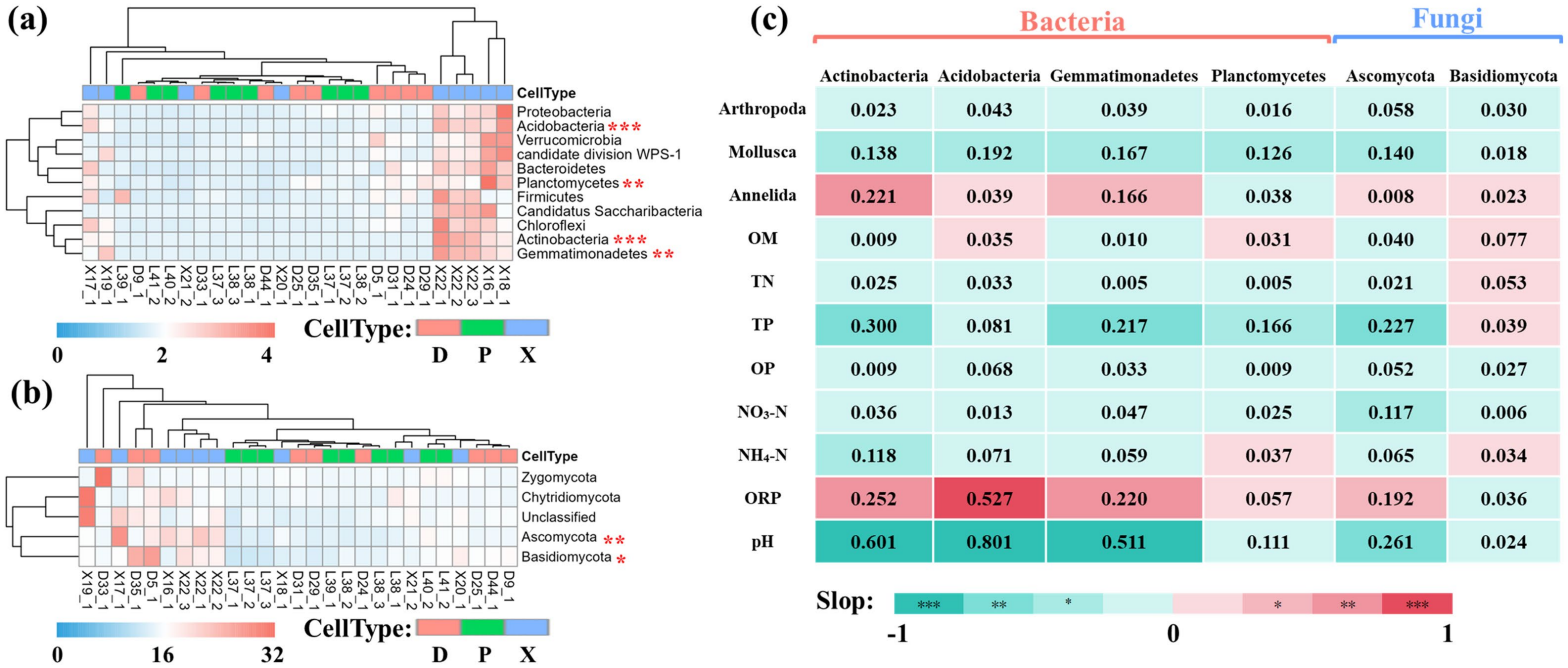
Heat map analysis of unique OTUs at phyla level. Heat map of core phyla (RAaverage >0.10%) of unique OTUs for bacterial (a) and fungal (b) communities in main Dongting Lake (D), Xiangjiang River (X), and pooling from three outfalls (P). Data were normalized by z-scores, and Euclidean distances were subjected to UPGMA cluster analysis. Phylum with significant differences among the three sections were selected by ANOVA and marked as follows: *p < 0.05, ** *p* < 0.01, and *** *p* < 0.001. Correlation heat map analysis of microbial phylum and biotic and abiotic variables (c). The correlation was performed by linear regression analysis, and R^2^ was labeled in the heatmap. The positive slope indicated a positive correlation and was labeled as red in the correlation heat map, while the negative slope indicated a negative correlation and was labeled as green.

We also evaluated the contributions of important biotic and abiotic factors to bacterial and fungal community composition via the results of multivariate regression analysis. We first selected 11 and 4 core phyla of unique OTUs for bacterial and fungal communities, respectively (RA_average_ > 0.10%) (Figures 6a,b). Then, ANOVA found that *Actinobacteria, Acidobacteria, Gemmatimonadetes,* and *Planctomycetes* showed significant differences among bacterial communities of D, P, and X, and *Ascomycota* and *Basidiomycota* had significant differences among fungal communities of D, P, and X (Tukey–Kramer tests, *p* < 0.05). Finally, linear regression analysis between these phyla and environmental factors was conducted (Figure 6c). Biotic factors *Mollusca* was found to be negatively related to the relative abundance of *Actinobacteria*, *Acidobacteria*, *Gemmatimonadetes*, *Planctomycetes*, and *Ascomycota* (*p* < 0.05), while *Annelida* was positively related to the relative abundance of *Actinobacteria* and *Gemmatimonadetes*. For abiotic factors, the relative abundance of *Actinobacteria*, *Acidobacteria*, *Gemmatimonadetes*, and *Ascomycota* increased with the increasing ORP and decreasing pH and TP. In addition, *Ascomycota* increased with decreasing NO_3_-N, but *Actinobacteria* increased with decreasing NH_4_-N.

### 3.4 Regulation of biotic and abiotic factors on microbial community functional diversity and composition

**Figure 7:**
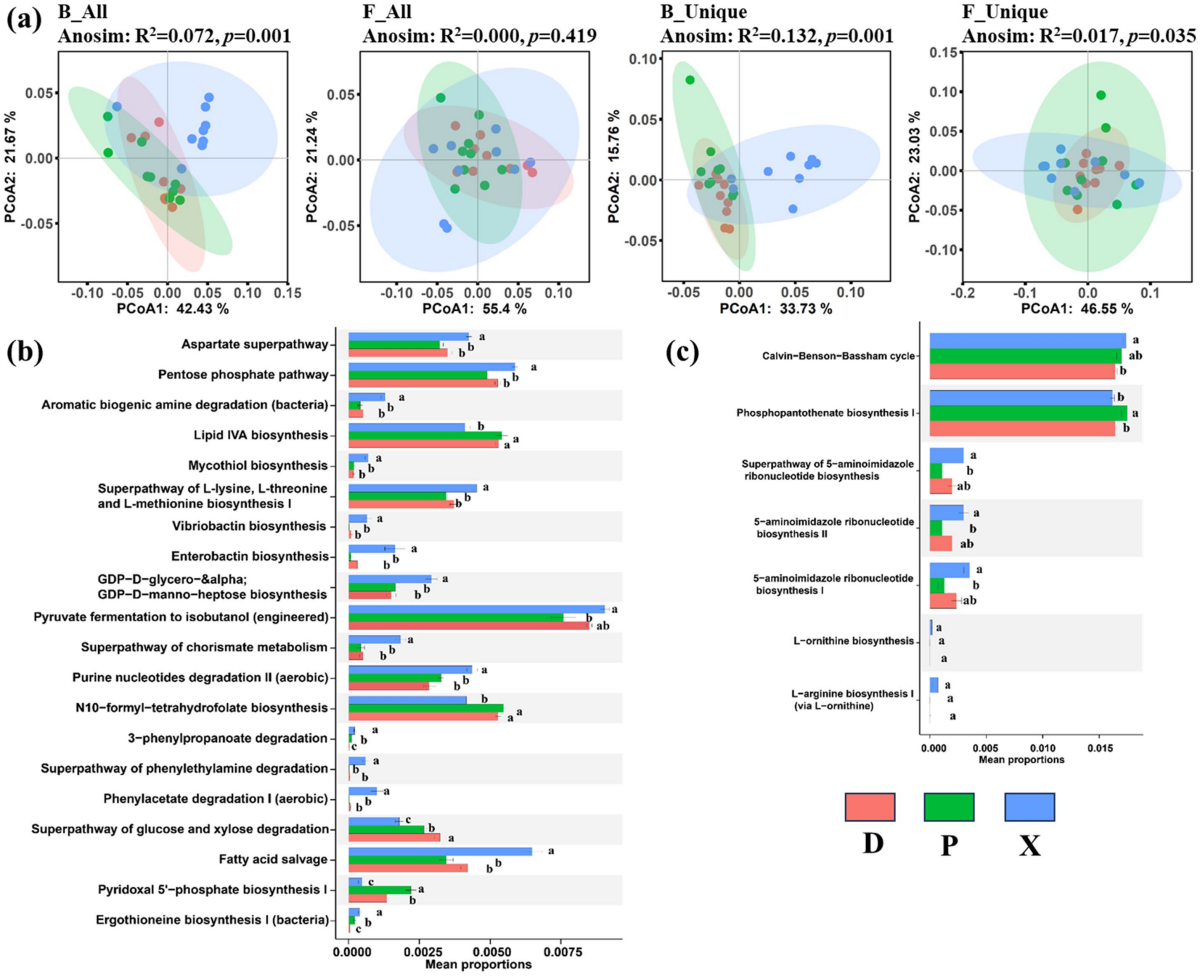
Function predicted for 16S rRNA and ITS by PICRUSt. PCoA analysis plots and ANOSIM analysis for the all and unique bacterial and fungal function composition among the main Dongting Lake (D), Xiangjiang River (X), and pooling from three outfalls (P) based on the Bray–Curtis distance similarity (a). Difference in functional pathway prediction among three sections for bacterial (b) and fungal (c) unique OTUs. Data were expressed as mean ± SD (*n* = 9). Different lowercase alphabet letters were significantly different at a *p*-value of <0.05.

We further analyzed the function types of bacterial and fungal communities for all OTUs and unique OTUs, and a significant difference was detected among the D, P, and X groups (ANOSIM, *p* < 0.05). The results of PCoA displayed that bacterial and fungal community functions were clustered into different groups according to different sections as shown in Figure 7a, particularly distinguishing between the X, D, and P sample clusters. Additionally, ANOSIM analysis revealed significant differences in B_All (R_2_ = 0.072, *p* = 0.001), B_unique (R_2_ = 0.132, *p* = 0.001), and F_all (R_2_ = 0.017, *p* = 0.035) community functions among the different sections. It is worth noting that bacterial and fungal community functions for unique OTUs exhibited higher dissimilarity than those for all OTUs. Furthermore, 20 and 7 significant differential pathways were detected in bacterial and fungal community functions for unique OTUs, respectively (Figures 7b,c). According to the KEGG database, amino acid metabolism pathway [including aspartate superpathway, aromatic biogenic amine degradation (bacteria), mycothiol biosynthesis, superpathway of L-lysine, L-threonine, and L-methionine biosynthesis I, superpathway of chorismate metabolism, superpathway of phenylethylamine degradation, phenylacetate degradation I (aerobic), and ergothioneine biosynthesis I (bacteria)], carbohydrate metabolism pathway (pentose phosphate pathway), metabolism of terpenoids and polyketides pathway (vibriobactin biosynthesis), lipid metabolism (including enterobactin biosynthesis and fatty acid salvage), glycan biosynthesis and metabolism pathway (GDP-D-glycero−&alpha/GDP-D-manno-heptose biosynthesis), nucleotide metabolism pathway [purine nucleotides degradation II (aerobic)], and xenobiotics biodegradation and metabolism pathway (3-phenylpropanoate degradation) were significantly enriched in bacterial communities of the X samples compared to the D and P samples (ANOVA, *p* < 0.05), while the functional types of glycan biosynthesis and metabolism pathway (lipid IVA biosynthesis), metabolism pathway of cofactors and vitamins (including N10-formyl-tetrahydrofolate biosynthesis and pyridoxal 5′-phosphate biosynthesis I), and biosynthesis of other secondary metabolites pathway (superpathway of glucose and xylose degradation) were enriched in the D samples compared to the X samples. Meanwhile, the functional types of energy metabolism (Calvin−Benson−Bassham cycle) and metabolism pathway of cofactors and vitamins (phosphopantothenate biosynthesis I) were enriched in fungal communities of the X or P samples compared to the D samples.

## 4 Discussion

This study comprehensively investigated the sediment environment characters and microbial community composition, diversity, and function types of bacteria and fungi in Dongting Lake (D) and its tributary rivers, including Xiangjiang River (X), and pooling from three outfalls (P). The results revealed significant differences in sediment properties and zoobenthos community compositions among the main lake and tributary rivers, indicating distinct biotic and abiotic environmental characteristics. The main lake had higher nutrient contents than tributary rivers. In detail, the contents of TP, NO^3−^-N, and NH^4+^-N were higher in D than those in X, while OM was higher in D than that in P. In a previous study, water inputs from rivers can alter the dissolved oxygen, conductivity, turbidity, and nutrient levels, among other variables, in lakes (Weigelhofer et al., 2015; Castillo, 2020). Lake sediments may permanently bury 70% of nutrient-enriched particulate nutrients derived from tributaries, rather than releasing them into the environment (Klump et al., 1997). For example, nutrient inputs such as phosphorus and nitrogen in Lake Erie originated from the Maumee River (Stow et al., 2015). Thus, sediment nutrient in lakes was considered to be higher than that in tributary rivers. It was typically found that river mainstems have higher concentrations of nitrate than lakes and backwaters, where depletion of nitrates is common (Houser, 2016). In addition, lower turbulence in lakes would further promote the enrichment of nutrient elements and reduce their release effect to overlying water compared to tributary rivers. Meanwhile, connections between rivers and lakes were crucial to determining macrozoobenthos assemblages (Pan et al., 2011). For example, tributaries serve as the main richness source for the Oligochaeta assemblage (Annelida) of the Neotropical dammed river (Ragonha et al., 2014). The present study showed that macrozoobenthos communities in Dongting Lake and its tributary rivers were composed of *Mollusca*, *Arthropoda*, and *Annelida*. *Arthropoda* and *Mollusca* were the dominant phyla in D, where tributary river P was dominated by *Mollusca*, and tributary river X was dominated by *Arthropoda* and *Annelida*. The Benthos macroinvertebrate community in the main Dongting Lake was identified to be higher than that in its tributary rivers under the action of species communication in the river–lake continuum (Roesti et al., 2015). Aquatic environment characteristics, including water physical and chemical variables (e.g., dissolved oxygen, conductivity, alkalinity, and temperature), land use, and landscape characteristics, are essential to understanding macroinvertebrate community distribution and diversity (Melo, 2009; Rezende et al., 2014). Environmental heterogeneity (e.g., nutrient levels and composition, hydrological conditions) between the main lake and tributary rivers in the present study drove the community differences in benthic macrofauna (Mao et al., 2023). Water velocity also affects benthic taxa through their respiration and feeding strategies (Hamid et al., 2021). *Chironomids* (*Arthropoda*) and *Oligochaetes* (*Annelida*) are generally tolerant of high nutrient concentrations and low oxygen levels and can outcompete sensitive taxa such as *Mollusca* (Brauns et al., 2007).

The comprehensive analysis of sediment microbial community composition, diversity, and distribution patterns across Dongting Lake and its tributary rivers provides valuable insights into the ecological dynamics of aquatic microbes in the river–lake continuum. The majority of microbes generally existed in both the lake and its tributary rivers under the exchanges of water and sediment in the river–lake continuum, with more than 50% sharing OTUs in bacterial and fungal communities. It is evident that the microbes and suspended matter entering the aquatic ecosystem from runoff are mixed with the microbial population inhabiting river and lake sediments. However, bacterial and fungal community dissimilarities were significantly different among the main lake and its tributary rivers, with the main effect of species replacement processes. On the one hand, Xiangjiang River consisted of more unique OTUs with the main lake compared to the pooling from three outfalls, which suggested a higher similarity of microbial community between the main Dongting Lake and the three outfalls. The three outfalls served as important exchange channels between Dongting Lake and Yangtze River, providing more than 30% of the runoff volume and 70% of the sediment load to Dongting Lake (Yu et al., 2018; Tian et al., 2021). The Xiangjiang River drains an area of 94,000 km^2^ and mainly derives its water from rainfall, flowing northward to Dongting Lake from the South Mountain Range (Xie et al., 2021). Microorganisms are transported together with particulate organic matter from one habitat to another, leading to microbial community coalescence due to the connection between the river and the lake (Bourhane et al., 2022). The dissimilarity of microbial communities between the Xiangjiang River and the main Dongting Lake was higher than that between the pooling from three outfalls and the main lake, due to the differences in hydrology conditions. On the other hand, the bacterial community had lower species richness in the Xiangjiang River than that in the main lake and the three outfalls, while the fungal community displayed reversed results. The dissimilarity of fungal communities between Dongting Lake and its tributary rivers was higher than that for the bacterial community. There are several possible explanations for this result. First, there are a variety of differences among bacteria and fungi characteristics, including growth rate and dispersal capacity, which may affect the assembly of benthic microbial communities and their biogeographic patterns in lake sediments (Zhao et al., 2022). A large proportion of fungal spores spread over short distances, which reduced the fungi exchanges among different habitats. The fungal community finally showed higher diversity in the Xiangjiang River than in the main lake and the pooling from three outfalls. Then, benthic bacterial communities may be better able to utilize simple compounds in the sediments, which will allow them to establish diversity more rapidly than fungal communities (Santonja et al., 2018). In other words, bacteria would be more tolerant to environmental disturbances compared to fungi.

Biotic and abiotic factors in sediment habitat drive the contrast diversity patterns and processes of the microbial community among Dongting Lake and its tributary rivers by regulating the community’s unique species. Lakes and rivers were typically subjected to different environmental conditions, such as flow velocity, water residence time, organic matter quantity and quality, and nutrient content, which can affect the compositions and functions of the microbial community. PH, TP, and ORP were found to be the important abiotic variables for predicting the bacterial community structure, yet, pH, TP, and OP were key regulators for fungal community structure. Sediment pH dominated the biogeographic pattern of benthic bacterial and fungal communities in main lakes and rivers, in agreement with previous studies (Zhao et al., 2022). Sediment pH serves as an indicator of sediment condition as well as a key factor in the biogeographic distribution of benthic microbes (Hou et al., 2020). As most microbes have an intracellular pH close to neutral and their optimum pH range is narrow, bacteria grow slowly to enhance stress resistance during soil pH fluctuations. In stressful environments, slow-growing species might not reach a high biomass but may remain healthy (Jousset et al., 2017). Thus, pH would regulate the microbial community structure by affecting the relative abundance of bacterial and fungal unique species. Similarly, *Mollusca* was a vital biotic variable for predicting sediment bacterial and fungal community structure by affecting the relative abundance of unique species. The bioturbation and feeding habits of macroinvertebrates might have a significant impact on the structure of microbial communities (Li et al., 2020). On one hand, *Mollusca* has been found to affect the microbial community diversity and the abundance of microbes that transform nitrogen. For example, the presence of Mollusca resulted in a sharp decrease in the phyla *Gemmatimonadetes, Actinobacteria, Acidobacteria, Plantomycetes, Chloroflexi, Firmicutes, Crenarcheota*, and *Verrucomicrobia* (Black et al., 2017). A similar result was found in this study that *Mollusca* would decrease the unique phyla *Actinobacteria, Acidobacteria, Plantomycetes*, and *Gemmatimonadetes* in the bacterial community and *Ascomycota* in the fungal community. On the other hand, the existence of multiple species may be maintained by biotic interactions (e.g., competition), which prevent microbes from finely partitioning niche axes with small population sizes under limited sediment resource conditions. Additionally, *Mollusca*, including *Mytilus edulis, Pecten maximus, Ostrea edulis, Modiolus modiolus*, and *Mya arenaria*, feed on suspensions and actively filter and retain particles in the surrounding water, including bacteria that are free-living and encapsulated in particles (Mudadu et al., 2021; Kijewska et al., 2023). In contrast, *Annelida* can influence the relative abundance of unique species to regulate the bacterial community structures in sediment. The phylum *Annelida* consists of segmented worms, including *earthworms*, *lugworms*, *ragworms*, and *leeches*, which primarily inhabit freshwater environments as either free-living or parasitic organisms (Wang et al., 2021). Their main ecological role is to rework sediment habitats. As some of the most sensitive organisms in sediment, bacteria, especially unique species in different sections of the river–lake continuum, were positively affected when the abundance of *Annelida* changed, which was opposite to the effects observed with *Mollusca*.

Metabolic pathway predictions are essential for describing the metabolic activity of microbes (Liu et al., 2021). The microbial functional types were significantly different among Dongting Lake and its tributary rivers with the changes in microbial communities. First, the functional dissimilarity among lakes and rivers was strengthened in unique species compared to all species in bacterial and fungal communities. The result indicated that the unique species replacement in specific habitats would help to enhance some specific function types as an adaptation strategy in varying environments. Then, the results of our PICRUSt2 prediction approach showed that changes in unique species generally affected the metabolism functions of bacterial and fungal communities, which is consistent with previous studies (Yang et al., 2022). The main difference for bacterial functional types is that amino acid metabolism and lipid metabolism were enriched in the Xiangjiang River, and the metabolism of cofactors and vitamins was enriched in the main Dongting Lake. In addition, the fungal function types of carbohydrate metabolism and metabolism of cofactors and vitamins were also enriched in tributary rivers. The pentose phosphate pathway is an alternative to glycolysis, allowing microorganisms to metabolize a greater variety of carbohydrates without oxygen (Wamelink et al., 2008). A higher abundance of pentose phosphate pathway in the Xiangjiang River meant that microbes might have a stronger potential to mineralize a greater diversity of organic carbon substrate than those in the main Dongting Lake. Pyridoxal 5′-phosphate is a B6 vitamer, which regulates several metabolic processes, including several metabolic reactions, such as amino acid biosynthesis and degradation, iron metabolism, nucleotide utilization, cofactor biosynthesis, and biofilm formation (Kuo et al., 2023). The lipid IVA biosynthesis is one of the main conserved structures in diverse Gram-negative pathogens, which is the most toxic part (Amin et al., 2023).

## 5 Conclusion

Although the main Dongting Lake and its tributary rivers are parts of a lake–river continuum system, there are significant differences in the biotic and abiotic environments of their sediment habitats. The value of pH was found to be the most important abiotic variable for predicting the bacterial and fungal community structures, and the abundance of *Mollusca* was found to be the most important biotic variable. The bacterial and fungal community compositional dissimilarities among Dongting Lake, Xiangjiang River, and pooling from three outfalls were dominated by species replacement processes. In particular, the biotic and abiotic factors affected the number and the relative abundance of bacterial and fungal unique OTUs and resulted in the changes in community composition for these unique OTUs. *Mollusca*, pH, TP, NO_3_-N, and NH_4_-N were negatively related to the relative abundance of *Actinobacteria, Acidobacteria, Gemmatimonadetes, Planctomycetes*, and *Ascomycota*, while *Annelida* and ORP were positively related to the relative abundance of *Actinobacteria* and *Gemmatimonadetes*. Finally, the functional dissimilarity between lakes and rivers was strengthened in unique species compared to all species in bacterial and fungal communities, which helped to improve the habitat environment in main Dongting Lake and promote the process of microbial growth. Therefore, the results of this study provided a theoretical reference for further understanding of microbial community structure, function, and the key influencing factors in lake–river continuum sediments.

## Data Availability

The datasets presented in this study can be found in online repositories. The names of the repository/repositories and accession number(s) can be found at: https://www.ncbi.nlm.nih.gov/, PRJNA801141; https://www.ncbi.nlm.nih.gov/, PRJNA801493.

## Footnotes

^1^ http://ieg4.rccc.ou.edu/index.cgi

^2^ https://www.omicstudio.cn/analysis/
