# Supplementary material for: Biotic and abiotic properties mediating sediment microbial diversity and function in a river–lake continuum
Source: Front Microbiol. 2024 Oct 21;15:1479670. doi: 10.3389/fmicb.2024.1479670 (PMC11532113; doi:10.3389/fmicb.2024.1479670)
Supplement: Supplementary file 1 [file Table_1.docx]

## Biotic and abiotic properties mediating sediment microbial diversity and function patterns in a river-lake continuum

Yabing Gu^a, b, c^, Delong Meng^c^, Zhenghua Liu^c^, Min Zhang^c^, Zhaoyue Yang^c^, Huaqun Yin^c^, Yanjie Liang^a^, **Nengwen Xiao^b*^**

^a^ School of Metallurgy and Environment, Central South University, Changsha, 410083, China;

^b^ State Key Laboratory of Environmental Criteria and Risk Assessment, Chinese Research Academy of Environmental Sciences, Beijing, 100012, China;

^c^ School of Minerals Processing and Bioengineering, Central South University, Changsha, 410083, China;

* Corresponding author:

Nengwen Xiao, xiaonw@163.com.

E-mail:

Yabing Gu: [guyabing0207@163.com](mailto:guyabing0207@163.com);

Delong Meng: [delong.meng@csu.edu.cn](mailto:delong.meng@csu.edu.cn);

Zhenghua Liu: liuzhenghua2017csu@163.com;

Min Zhang: [205606002@csu.edu.cn](mailto:205606002@csu.edu.cn);

Zhaoyue Yang: yangzhaoyue@csu.edu.cn

Huaqun Yin: [yinhuaqun_cs@sina.com](mailto:yinhuaqun_cs@sina.com);

Yanjie Liang, LiangyanjieCSU@163.com;

Nengwen Xiao: [xiaonw@craes.org.cn](mailto:xiaonw@craes.org.cn).

**Table. S1** Description of the 27 sampling sites and their geographical distance.

| **Number** | **Site** | **Longitude** | **Latitude** |
| --- | --- | --- | --- |
| 1 | DS5_1 | 112.896 | 29.429 |
| 2 | DS9_1 | 113.058 | 29.338 |
| 3 | DS16_1 | 112.969 | 29.08 |
| 4 | DS17_1 | 112.945 | 29.02 |
| 5 | DS18_1 | 113.037 | 28.88 |
| 6 | DS19_1 | 112.809 | 28.568 |
| 7 | DS20_1 | 112.861 | 28.706 |
| 8 | DS21_1 | 112.838 | 28.765 |
| 9 | DS22_1 | 112.894 | 28.827 |
| 10 | DS22_2 | 112.894 | 28.827 |
| 11 | DS22_3 | 112.894 | 28.827 |
| 12 | DS24_1 | 112.28 | 28.855 |
| 13 | DS25_1 | 112.296 | 28.841 |
| 14 | DS29_1 | 112.399 | 28.851 |
| 15 | DS31_1 | 112.426 | 28.804 |
| 16 | DS33_1 | 112.517 | 28.808 |
| 17 | DS35_1 | 112.559 | 28.857 |
| 18 | DS37_1 | 112.254 | 29.103 |
| 19 | DS37_2 | 112.254 | 29.103 |
| 20 | DS37_3 | 112.254 | 29.103 |
| 21 | DS38_1 | 112.287 | 29.063 |
| 22 | DS38_2 | 112.287 | 29.063 |
| 23 | DS38_3 | 112.287 | 29.063 |
| 24 | DS39_1 | 112.305 | 29.07 |
| 25 | DS40_1 | 112.317 | 29.054 |
| 26 | DS41_1 | 112.334 | 29.045 |
| 27 | DS44_1 | 112.211 | 28.916 |
